# The impact of innate and humoral immune mechanisms on vaccine induced protection against avian influenza H9N2 in broilers

**DOI:** 10.1038/s41598-025-28122-2

**Published:** 2025-12-02

**Authors:** Hesham M. Asaad, Ola Hassanin, Mustafa Saif-Edin, Ragab S. Ibrahim, Moemen A. Mohamed, Mustafa Hamad, Tamer Mahmoud Abdullatif

**Affiliations:** 1 Poultry Diseases Department, Faculty of Veterinary Medicine, Sohag University, Sohag, Egypt; 2 Avian and Rabbit Medicine Department, Faculty of Veterinary Medicine, Zagazig University, Zagazig, Egypt; 3 Avian and Rabbit Medicine Department, Faculty of Veterinary Medicine, Assiut University, Assuit, Egypt; 4 School of Veterinary Medicne, Badr University, Assuit Branch, Assiut, Egypt

**Keywords:** Chicken, Avian influenza, H9N2, Vaccine, Cytokine, Interferon, Vaccines, Virology

## Abstract

Avian influenza virus (AIV) (H9N2) is a highly contagious avian respiratory disease that causes significant economic losses in the poultry industry sector. Although immunological responses to AIV (H9N2) vaccinations have been extensively studied, little is known about how inactivated vaccines interact with the host’s innate and adaptive immune responses. Five commercially available inactivated avian influenza virus (AIV) H9N2 vaccines, each containing different virus strains and adjuvant compositions, were administered via subcutaneous injection to 4-day-old commercial broiler chicks. The effects of vaccination on the kinetics of chicken toll-like receptor (TLR) 5 and 7 pathways, along with their downstream cytokines and the expression profiles of major histocompatibility complex class I and 2 (MHC-1 and 2) expression profiles were investigated. Quantitative real-time PCR analysis demonstrated that immunization with inactivated H9N2 AIV vaccines activated pattern recognition receptors (PRRs), specifically TLR-5 and TLR-7, along with their downstream cytokines. These included the proinflammatory interleukin-6 (IL-6), antiviral interferon-alpha, and myxoma resistance protein 1 (Mx1). This activation was observed as early as 48 h post-vaccination and persisted for up to 7 days in the spleens of the vaccinated birds. Subcutaneous administration of either inactivated vaccine A or B elicited the highest expression levels of IL-6, MHC class I, and MHC-1 and MHC-2 molecules. Hemagglutination-inhibition (HI) antibody titers against avian influenza were > 5 log_2_ from two to five weeks post-vaccination, with vaccine A inducing significantly higher responses at weeks three, four, and five. Following intraocular challenge with 10^6^ EID_50_/ml of a locally isolated avian influenza virus strain, vaccines A, B, and E conferred complete (100%) protection against mortality. Notably, vaccine A demonstrated superior clinical protection and resulted in minimal histopathological lesions in the tracheal and pulmonary tissues.

**Supplementary Information:**

The online version contains supplementary material available at 10.1038/s41598-025-28122-2.

## Introduction

Respiratory infections have increased in Egyptian commercial poultry flocks over the past ten years^1^. Avian influenza virus is one of the most serious viral infections affecting poultry, with a global distribution that impacts various bird species^2^. Avian influenza viruses are classified into two categories: highly pathogenic avian influenza (HPAI) viruses, which cause severe and often fatal illnesses in chickens, and low pathogenic avian influenza (LPAI) viruses, which are significantly less virulent^3^. The H9N2 avian influenza virus subtype is known to be low pathogenic in chickens^4^; however, when it coexists with other infections, it can result in significant losses in the poultry industry^5^. Furthermore, this virus has the potential to transfer internal genes to HPAIV, which can lead to increased pathogenicity^6^. The H9N2 subtype of avian influenza viruses can be phylogenetically classified into two primary lineages: American and Eurasian. Within these two lineages, additional clusters can be identified due to the virus’s ongoing evolution. Prevention of the emergence of AIV H9N2 and co-epidemics is critically important. While various methods for disease prevention exist worldwide, including stringent biosecurity measures and the eradication of infected flocks^7,8^, vaccines play a crucial role in global disease prevention and control. Several types of AI vaccines have already been utilized, including inactivated vaccines^9,10^, reverse genetics vaccines^11^ and recombinant virus vector vaccines^12^. Nevertheless, LPAI (H9N2) viruses continue to pose a significant challenge for several reasons. The primary factors contributing to this issue include viral evolution, the emergence of new divergent strains, immunosuppressive diseases, interference from maternally derived antibodies, and deficiencies in the vaccination process^13–15^.

To enhance vaccine efficacy, the seed strain needs to possess genetic and antigenic characteristics that closely align with those of the circulating field viruses^16^. Additionally, the vaccination formulation should effectively elicit robust innate immune responses, which subsequently lead to adaptive immunity in avian hosts. Commercially available inactivated avian influenza virus (AIV) H9N2 vaccines vary in their seed strains, antigenic content, and the types of emulsified adjuvants used. Adjuvants, biological or chemical agents, serve as delivery systems for inactivated pathogens and can stimulate both innate and adaptive immune responses against specific pathogens when incorporated into vaccine formulations^7,17^.

This immunological activation followed AIV (H9N2) vaccination confers protection against morbidity, mortality, and declines in egg production associated with avian influenza^13,18,19^. Prior experimental studies have shown that adjuvant-based inactivated avian influenza vaccines are capable of eliciting innate immune responses, which are subsequently followed by the generation of humoral antibodies. A comprehensive study of those two arms of immune responses elicited post AIV (H9N2) immunization will help in understanding AIV pathogenesis, immunology and designing effective vaccine. The innate immune response is regarded as the first line of defense and represents the immediate immune reaction triggered by AIV (H9N2) immunization or infection. It initiates several signaling cascades upon activation by live or inactivated pathogens. These cascades involve various sensors in host cells known as pattern recognition receptors (PRRs), including Toll-like receptors (TLR-3, TLR-5, and TLR-7), Melanoma Differentiation-Associated gene 5 (MDA5), cyclic GMP-AMP synthase (cGAS), and members of the DDX family (DDX1 and DDX3X)^20,21^. These sensors interact with pathogen-associated molecular patterns (PAMPs), including viral single-stranded and double-stranded RNA. This interaction triggers signaling cascades that activate interferon regulatory factors 1 & 7 as well as the nuclear factor kappa-light-chain-enhancer of activated B cells (NF-κB), resulting in the induction of chicken type I interferons. Type I interferons bind to their corresponding receptors on target cells, initiating the JAK-STAT signaling pathway. Stimulation of the JAK-STAT pathway culminates in an antiviral state characterized by the transcriptional activation of a variety of interferon-stimulated genes (ISGs), which confer antiviral response^22,23^. Pro-inflammatory cytokines, such as IL-6, are consistently synthesized in response to the recognition of PAMPs by TLRs, including TLR-7 and TLR-5, as well as the activation of the NF-κB promoter. These cytokines play crucial roles in regulating leukocyte activities and the synthesis of antimicrobial peptides and interferons (IFNs), which are essential for pathogen elimination. Both type I interferons, which are antiviral, and pro-inflammatory cytokines such as interleukin-1 beta (IL-1β) and IL-6 serve as primary mediators in the host immune response induced by AIV. These cytokines are primarily believed to mobilize the immune response and exert antiviral effects, thereby acting as regulators of the host’s immune response to infection^24^. In chickens, the combination of TLR ligands with inactivated AIV vaccines has been shown to effectively induce protective immune responses^9,25^.

The effectiveness of various inactivated H9N2 AIV vaccines in combating influenza infections primarily depends on their ability to modulate different immune pathway compartments in chickens. Consequently, the objective of this experiment was to conduct a comprehensive comparative analysis of various commercial H9N2 AIV vaccines, which differ in seed strains and emulsified adjuvants. This study investigates the subsequent production of innate antiviral and pro-inflammatory immune responses following vaccination, as well as the resulting humoral adaptive immune responses. A challenge experiment was performed to assess the level of clinical protection and resistance to infection in the vaccinated birds.

## Material and methods

### Birds

One hundred and eighty, one-day-old Cobb broiler commercial chicks (for study 1) and 90 one-day-old Cobb broiler commercial chicks (for study 2) were utilized. The birds were reared in a floor-based system in the animal house experimental facility at the Faculty of Veterinary Medicine, Zagazig University. The Zagazig University Institutional Animal Care and Use Committee (ZU-IACUC) approved the animal experiments with approval number (ZU-IACUC/2/F/92/2024). All animal protocols were carried out in compliance with the ARRIVE guidelines. All birds had ad libitum access to feed and water throughout the experimental period. The birds were monitored for any alterations that could indicate the presence of any infections. All the procedures were performed under high biosafety conditions that comply with local animal welfare regulation on experiments with chickens and similar species. Accordingly, all the animal euthanasia were performed in accordance with the American Veterinary Medical Association (AVMA) guidelines for animal euthanasia via cervical dislocation.

### Vaccines

#### AIV (H9N2) vaccines

Five commercial inactivated H9N2 vaccines were evaluated in this experiment, which were named as (A, B, C, D and E).

- Vaccine A (Nobilis H9N2 + ND P.) is manufactured by Merck Sharp & Dohme incorporation, Salamanca, Spain. Vaccine A is prepared from inactivated H9N2 (A/CK/UAE/415/99) and Newcastle Disease Virus (strain Clone 30) vaccine. It administrated as a single dose of 0.25 ml in the neck region.
- Vaccine B (MEFLUVAC™ H9 + ND7) is manufactured by MEVAC incorporation, Egypt. Vaccine B is prepared from inactivated H9N2 (A/ck/Egypt/ME/543 V/2016) and NDV recombinant GVII strain (rgNDV1/ME.G7/2017). It administrated as a single dose of 0.25 ml in the neck region.
- Vaccine C (CEVAC® NEW FLU H9 K) is produced by Ceva incorporation, Budapest, Hungary. Vaccine C is prepared from H9N2 (sub-type G1-like sub lineage of Middle East origin) and LaSota strain vaccine. It administrated as a single dose of 0.25 ml in the neck region.
- Vaccine D (GALLIMUNE 208 ND + FLU H9) is produced by Merial incorporation, Lyon, France. Vaccine D is prepared from H9N2 (A/chicken/Iran/Av1221/1998) and Ulster 2C strain vaccine. It administrated as a single dose of 0.25 ml in the neck region.
- Vaccine E (ValleyVac H9–ND^G7^) is manufactured by the Egyptian Company for Biological & Pharmaceutical Industries 101 extension of the sixth industrial zone-6^th^ of October City, Egypt. Vaccine E is prepared from H9N2 (A/chicken/Egypt/S10490/2015) and NDV GVII strain vaccine. It administrated as a single dose of 0.25 ml in the neck region.

#### Other vaccines

All birds were given vaccines for infectious bursal disease virus (IBDV), AIV-H5, infectious bronchitis virus (IBV) and Newcastle disease virus (NDV).

- Infectious bursal disease (IBD) vaccine is manufactured by Lohmann Company, Germany, manufactured for Elanco. The vaccine is prepared from intermediate (LC-75) vaccine strain and administered by eye drop route to 10 and 20 days old birds.
- Avian influenza vaccine (HPAI) is produced by Pulike (Nj) Biological Technology Company Nanjing, China. The vaccine is prepared from H5N1 subtype (Re-5) vaccine strain and administered subcutaneously at a dose of 0.3 ml/bird to 7 days old chicks.
- Infectious bronchitis (IB) and Newcastle disease (ND) live freeze dried vaccine is produced by Ceva incorporation, Budapest, Hungary. The Vaccine is prepared from IB (Massachusetts H120) and (PHY. LMV.42) strain vaccine and administered by eye drop route to 7 days old chicks.
- Newcastle Disease (ND) Vaccine is manufactured by Merck Sharp & Dohme incorporation, Salamanca, Spain. The vaccine is prepared from a clone selected LaSota strain, B1 type Newcastle disease virus and administered by eye drop route to 17 days old chicks.

### Challenge virus

Avian influenza virus H9N2 G1 lineages (A/chicken/Egypt/FAO-S33/2021 (H9N2)) with an accession number of (ok148893) were used as challenge virus. The infective dose was adjusted to contain 10^6^ embryo infective dose 50 (EID_50_) / ml and the birds were challenged via the ocular route.

### Experimental design

#### Study 1: innate and humoral immune responses conferred post vaccination with different commercial inactivated AIV (H9N2) vaccines

–One hundred and eighty, one-day-old broiler chicks were divided into six groups (n = 30) as shown in (Table 1). Different bird groups received the following; Nobilis H9N2 + ND P. (Vaccine A), MEFLUVAC ™ H9 + ND7 (Vaccine B), CEVAC® NEW FLU H9 K (Vaccine C), Gallimune Flu H9 M.E. (Vaccine D) and ValleyVac H9–ND^G7^ (Vaccine E). These vaccines were administered subcutaneously to 4 days old chicks according to the dose recommended by the manufacturer and the ages of vaccination are listed in Table 1. Finally, the sixth group was considered as negative control group (non-vaccinated).

**Table 1:** Experimental design of differentially vaccinated groups.

Groups	Vaccines				
	H9N2 Vaccine At 4 days old chicks	Other vaccines			
		IB Vaccine At 7 days old	H5N1Vaccine At 7 days old	IBD Vaccine At 10 and 20 days old	ND Vaccine At 17 days old
Vaccine A	Vaccine A	+	+	+	+
Vaccine B	Vaccine B	+	+	+	+
Vaccine C	Vaccine C	+	+	+	+
Vaccine D	Vaccine D	+	+	+	+
Vaccine E	Vaccine E	+	+	+	+
Control (non-vaccinated)	–	+	+	+	+

All of the birds were vaccinated against Newcastle disease virus, infectious bursal disease virus, H5 virus and infectious bronchitis virus at the defined time and by the appropriate route of vaccination as shown in (Table 1**)**. After euthanasia by cervical dislocation, spleen samples (n = 5 per group) were collected on days 1, 2, 3, and 7 following vaccination. The samples were immersed in RNA*later*™ at a 5 × concentration, incubated for 24 h at 4 °C, and then stored at -80 °C. Blood samples were collected from the wing vein (n = 10/group) at 1^st^, 2^nd^, 3^rd^, 4^th^ and 5^th^ week post vaccination and serum was separated and stored at -20 °C.

#### Study 2: Clinical protection of vaccinated chickens post challenge with AIV (H9N2) virus of Egyptian origin

Ninety, one-day-old broiler chicks were divided into six groups (n = 15) and vaccinated with the same protocol and organization of study 1. On 21 day of age, the birds were challenged with 10^6^ EID_50_/ml of AIV strain (A/chicken/Egypt/FAO-S33/2021 (H9N2)) via intraocular route. Birds were observed daily for 7 days for their clinical signs of disease and mortality and all observations were recorded. Tissues from lung and trachea were collected from three birds/group, one-week post challenge, for histopathological study. The lesions were graded by estimating by semiquantitative methods “Ordinal Method “in the entire sections. Lesions score system was as follows: 0 = absence of lesion, 1 = mild, 2 = moderate, 3 = severe and 4 = highly severe.

### RNA extraction, cDNA synthesis and quantitative real-time RTPCR

Spleen samples (20 mg) were collected (n = 5/group) at intervals of 1, 2, 3 and 7 days post vaccination with inactivated H9N2 vaccines and were immersed in RNA*later*™ at a 5 × concentration, incubated for 24 h at 4 °C, and then stored at -80 °C. Extraction of total RNA was performed according to the instructions of the ABT viral RNA extraction kit (Applied Biotechnology, Ismailia, Egypt). The purified RNA from each sample was reverse transcribed to cDNA using ABT H-minus cDNA synthesis kit (Applied Biotechnology, Ismailia, Egypt) and oligo dT 18 primer according to the manufacturer’s instructions. Quantitative Real time PCR was done using Applied Biosystems 7500 FAST Real Time PCR instrument and ABT 2X qPCR Mix (SYPR)-Low Rox, using the designed primer pairs targeting the studied cytokines, purchased from (Metabion), as described in (Table 2)^26,27^. The thermo profile was; 95 °C for 10 min hold, followed by 40 cycles of 95 °C for 10 s and 60 °C for 30 s. A melting curve was carried out to detect whether there was any non-specific amplification at 95 °C for 15 s, 60 °C for 1 min, 95 °C for 15 s and 60 °C for 15 s. GABDH was used as an internal control.

**Table 2:** Oligonucleotide primers used for the amplification of the different cytokines.

Cytokine	Forward primer	Reverse primer
TLR5	F5′-TCA AAG ATG GGT GGT GTG TAG AA-3′	R5′-ACT GAC GTT CCT TTG CAC TTT TT-3′
TLR7	F5′-ATG CTG TTA TCA GGA CGT TGG TT-3′	R5′-CCT TGA GGC GAC GGT CAC T-3′
MHC-1	F5′-AAG AAG GGG AAG GGC TAC AA-3′	R5′-AAG CAG TGC AGG CAA AGA AT-3′
MHC-2	F5′-CTC GAG GTC ATG ATC AGC AA-3′	R5′-TGT AAA CGT CTC CCC TTT GG-3′
IL6	F5′-GCG AGA ACA GCA TGG AGA TG-3′	R5′-GTA GGT CTG AAA GGC GAA CAG-3′
Interferon-Alpha	F5′-CCA GCA CCT CGA GCA AT-3′	R5′-GGC GCT GTA ATC GTT GTC T-3′
Mx1	F5′-AAC GCT GCT CAG GTC AGA AT-3′	R5′-GTG AAG CAC ATC CAA AAG CA-3′
GAPDH	F5′- CCT CTC TGG CAA AGT CCA AG -3′	R5′-CAT CTG CCC ATT TGA TGT TG-3′

### Serology

The hemagglutination inhibition (HI) assay was conducted using 4 hemagglutinin (HA) units of AIV strain A/chicken/Egypt/FAO-S33/2021(H9N2). In brief, ten serum samples from each group were heat-inactivated at 56 °C for 30 min. Then, 25 μl of the inactivated chicken sera were subjected to twofold serial dilutions in phosphate-buffered saline. A constant amount of antigen containing 4 HA units of the AIV strain was added to each dilution. After incubating for 15 min, 50 μl of 1% chicken red blood cells were added and incubated for an additional 20 min before the results were read. The HI titer was determined by identifying the last well that showed inhibition of hemagglutination. Titers were reported as log_2_ geometric mean titers (GMT).

### Histopathology

Lung and tracheal samples were collected for histopathological study. Specimens from each group were fixed at 10% neutral buffered formalin and were dehydrated in a graded alcohol series, cleared with xylene, embedded in paraffin wax, sectioned at 4–5 um thickness and stained with hematoxylin and eosin for histopathological examination by light microscopy^28^. Stained tissue sections were examined by light microscopy (Olympus, Japan) and photographed using digital camera (Olympus, Japan).

### Statistical analysis

Data were edited in Microsoft Excel (Microsoft Corporation). For serology analyses, one-way ANOVA was used, followed by Tukey’s Honestly Significant Difference (Tukey’s HSD) test as a post hoc test. The results were reported as mean ± SEM (Standard Error of Mean), with statistical significance set at 0.05. The gene expression levels were compared between groups via T-test. GraphPad Prism version 8 for Windows, GraphPad Software, La Jolla, California, USA, www. graphpad. com, was used to graph the data.

## Results

### Study 1: innate and humoral immune responses conferred post vaccination with different commercial inactivated AIV (H9N2) vaccines

#### Effect of different commercially available inactivated H9N2 vaccines on the kinetics of chicken TLR-5 & -7 pathways and their related down-stream cytokines

**Fig. 1:**
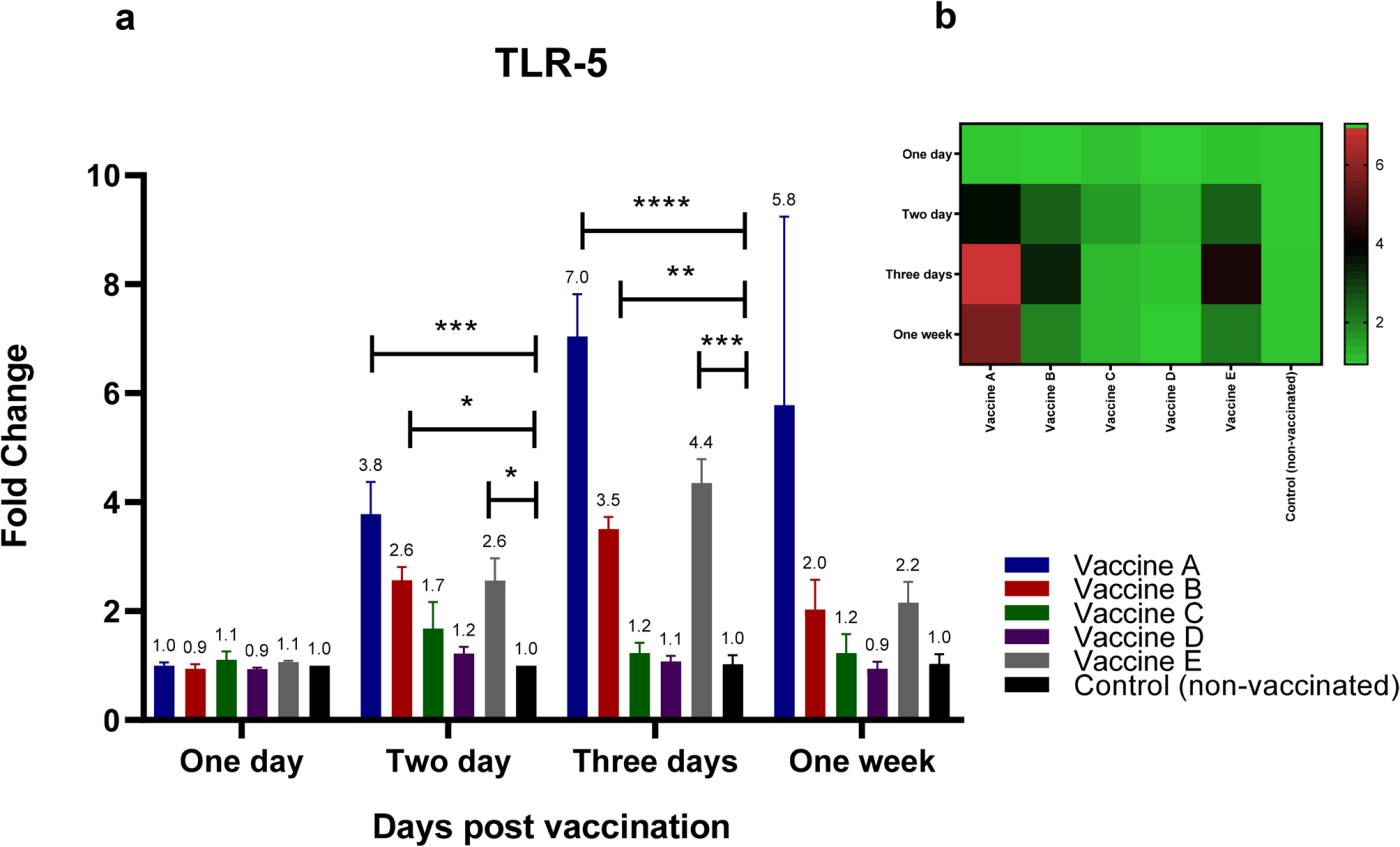
Effect of different commercially available inactivated H9N2 vaccines on the kinetics of chicken TLR-5 & -7 pathways and their related down-stream cytokines (TLR-5). Five different commercial AIV (H9) inactivated vaccines were administrated to different experimental groups 4 days of age. Five birds from each group were humanly euthanized 24 h, 48 h, 72 h and one-week post vaccination and their spleen tissues were collected. RNA was extracted and TLR-5 specific quantitative real-time PCR was performed. All data were normalized to GAPDH as an endogenous control and all data were expressed as mean of fold difference relative to non-AIV (H9) vaccinated group, which is given an arbitrary value of 1. (**a**) Error bars represent the standard errors of means and One-way ANOVA was used for the statistical analysis with Dunnett’s test at *p* < 0.05 using GraphPad Prism version 8 for Windows, GraphPad Software, La Jolla California USA, www.graphpad.com. Asterisks indicate the statistically significant difference compared to the control non-vaccinated group (*p ˂ 0.05, ** *p* ˂ 0.01, *** *p* ˂ 0.001 and **** *p* ˂ 0.0001). (**b**) Heat map of fold changes generated by comparing TLR-5 expression in vaccinated groups to non-vaccinated group. The fold change is shown on a log_2_ scale, as shown beside the heat map and the different vaccine letter indicate different vaccinated group.

Five different vaccinated groups, along with a non-immunized control group, were assessed for their capacity to produce various cytokines, TLR-5, TLR-7, interleukin-6 (IL-6), chicken interferon-alpha (chIFN-alpha), and Myxovirus resistance 1 (Mx1), in spleen tissues at 1, 2, 3, and 7 days post-vaccination. As illustrated in Fig. 1a-b, birds vaccinated with vaccines A, B, and E exhibited elevated TLR-5 mRNA expression in the chicken spleen as early as 48 hpv. The increases were 3.8-, 2.6-, and 2.6-fold, respectively, with statistically significant differences (*p* < 0.05) compared to the control group . Three days post-vaccination, administration of vaccines A, B, or E resulted in increases in TLR-5 mRNA expression levels by sevenfold, 3.5-fold, and fivefold, respectively, with statistically significant differences (*p* < 0.05) compared to controls (Fig. 1a, b). One week after vaccination, TLR-5 transcript levels in the spleens of birds vaccinated with vaccine A increased by 5.8-fold, and by twofold with vaccines B or E, relative to the control group (Fig. 1a, b).

**Fig. 2:**
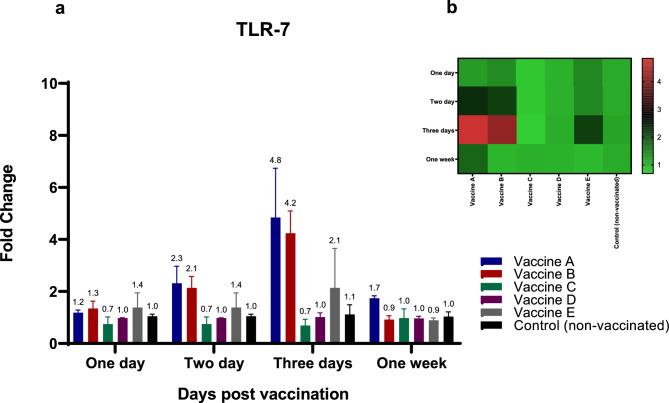
Effect of different commercially available inactivated H9N2 vaccines on the kinetics of chicken TLR-5 & -7 pathways and their related down-stream cytokines (TLR-7). Five different commercial AIV (H9) inactivated vaccines were administrated to different experimental groups 4 days of age. Five birds from each group were humanly euthanized 24 h, 48 h, 72 h and one-week post vaccination and their spleen tissues were collected. RNA was extracted and TLR-7 specific quantitative real-time PCR was performed. All data were normalized to GAPDH as an endogenous control and all data were expressed as mean of fold difference relative to non-AIV (H9) vaccinated group, which is given an arbitrary value of 1. (**a**) Error bars represent the standard errors of means and One-way ANOVA was used for the statistical analysis with Dunnett’s test at *p* < 0.05 using GraphPad Prism version 8 for Windows, GraphPad Software, La Jolla California USA, www.graphpad.com. Asterisks indicate the statistically significant difference compared to the control non-vaccinated group (*p ˂ 0.05, ** *p* ˂ 0.01, *** *p* ˂ 0.001 and **** *p* ˂ 0.0001). (**b**) Heat map of fold changes generated by comparing TLR-7 expression in vaccinated groups to non-vaccinated group. The fold change is shown on a log_2_ scale, as shown beside the heat map and the different vaccine letter indicate different vaccinated group.

Regarding chicken TLR-7, birds immunized with either vaccine A or B exhibited similar levels of TLR-7 stimulation, showing more than a twofold increase in spleen expression 48 hpv (Fig. 2a, b). Immunization with vaccines A, B, and E induced the highest host responses in the chicken spleen at 3 days post-vaccination, with 4.8-, 4.2-, and twofold increases, respectively, compared to the non-immunized control group (Fig. 2a, b). One-week post-vaccination, limited upregulation of TLR7 mRNA expression was observed only in birds that received vaccine A.

**Fig. 3:**
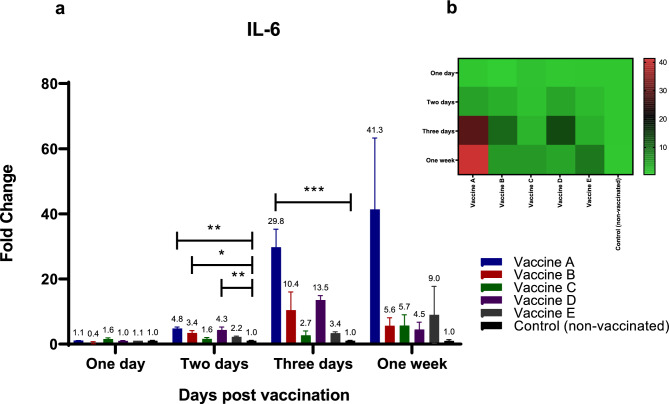
Effect of different commercially available inactivated H9N2 vaccines on the kinetics of chicken TLR-5 & -7 pathways and their related down-stream cytokines (IL-6). Five different commercial AIV (H9) inactivated vaccines were administrated to different experimental groups 4 days of age. Five birds from each group were humanly euthanized 24 h, 48 h, 72 h and one-week post vaccination and their spleen tissues were collected. RNA was extracted and IL-6 specific quantitative real-time PCR was performed. All data were normalized to GAPDH as an endogenous control and all data were expressed as mean of fold difference relative to non-AIV (H9) vaccinated group, which is given an arbitrary value of 1. (**a**) Error bars represent the standard errors of means and One-way ANOVA was used for the statistical analysis with Dunnett’s test at *p* < 0.05 using GraphPad Prism version 8 for Windows, GraphPad Software, La Jolla California USA, www.graphpad.com. Asterisks indicate the statistically significant difference compared to the control non-vaccinated group (*p ˂ 0.05, ** *p* ˂ 0.01, *** *p* ˂ 0.001 and **** *p* ˂ 0.0001). (**b**) Heat map of fold changes generated by comparing IL-6 expression in vaccinated groups to non-vaccinated group. The fold change is shown on a log_2_ scale, as shown beside the heat map and the different vaccine letter indicate different vaccinated group.

For IL-6 **(**Fig. 3a, b**)**, at 48 hpv, all immunized groups receiving different inactivated H9N2 vaccines exhibited increased IL-6 mRNA expression in chicken spleens to varying degrees, ranging from 1.6- to 4.7-fold increases. Statistically significant differences (*p* < 0.05) were observed only in the groups vaccinated with vaccines A, B, and D compared to the non-vaccinated control birds. Three days post-vaccination, immunization with vaccine A induced the highest response, with a 30-fold increase, while vaccines B, C, D, and E induced 10.4-, 2.7-, 13.5-, and 3.3-fold increases, respectively, compared to the control group **(**Fig. 3a, b**)**. Consistent with these results, at 7 dpv, vaccination with vaccine A led to the highest response (41.3-fold increase), while vaccines B, C, D, and E induced 5.6-, 5.7-, 4.5-, and ninefold increases, respectively, compared to the control group **(**Fig. 3a, b**)**.

**Fig. 4:**
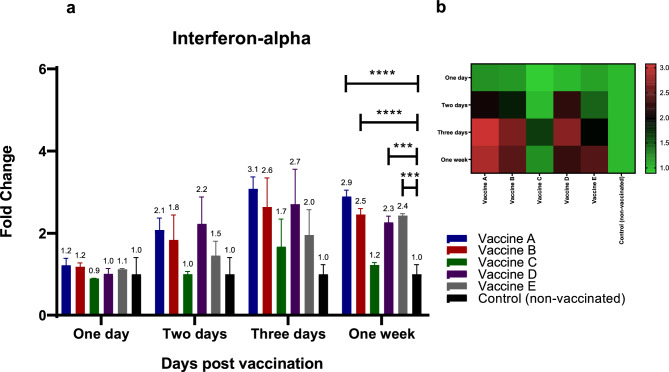
Effect of different commercially available inactivated H9N2 vaccines on the kinetics of chicken TLR-5 & -7 pathways and their related down-stream cytokines (chIFN-alpha). Five different commercial AIV (H9) inactivated vaccines were administrated to different experimental groups 4 days of age. Five birds from each group were humanly euthanized 24 h, 48 h, 72 h and one-week post vaccination and their spleen tissues were collected. RNA was extracted and chIFN-alpha specific quantitative real-time PCR was performed. All data were normalized to GAPDH as an endogenous control and all data were expressed as mean of fold difference relative to non-AIV (H9) vaccinated group, which is given an arbitrary value of 1. (**a**) Error bars represent the standard errors of means and One-way ANOVA was used for the statistical analysis with Dunnett’s test at *p* < 0.05 using GraphPad Prism version 8 for Windows, GraphPad Software, La Jolla California USA, www.graphpad.com. Asterisks indicate the statistically significant difference compared to the control non-vaccinated group (*p ˂ 0.05, ** *p* ˂ 0.01, *** *p* ˂ 0.001 and **** *p* ˂ 0.0001). (**b**) Heat map of fold changes generated by comparing chIFN-alpha expression in vaccinated groups to non-vaccinated group. The fold change is shown on a log_2_ scale, as shown beside the heat map and the different vaccine letter indicate different vaccinated group.

**Fig. 5:**
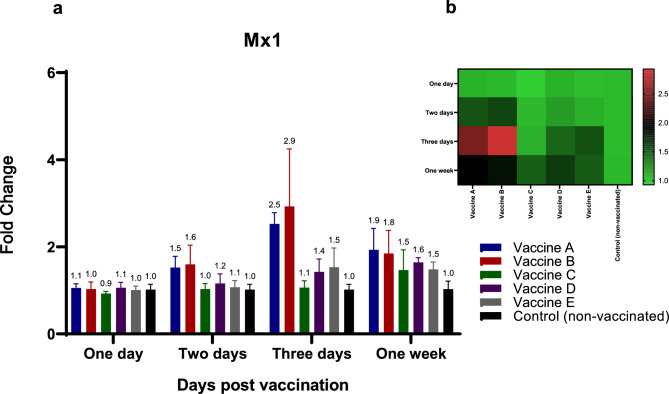
Effect of different commercially available inactivated H9N2 vaccines on the kinetics of chicken TLR-5 & -7 pathways and their related down-stream cytokines (Mx1). Five different commercial AIV (H9) inactivated vaccines were administrated to different experimental groups 4 days of age. Five birds from each group were humanly euthanized 24 h, 48 h, 72 h and one-week post vaccination and their spleen tissues were collected. RNA was extracted and Mx1 specific quantitative real-time PCR was performed. All data were normalized to GAPDH as an endogenous control and all data were expressed as mean of fold difference relative to non-AIV (H9) vaccinated group, which is given an arbitrary value of 1. (**a**) Error bars represent the standard errors of means and One-way ANOVA was used for the statistical analysis with Dunnett’s test at *p* < 0.05 using GraphPad Prism version 8 for Windows, GraphPad Software, La Jolla California USA, www.graphpad.com. Asterisks indicate the statistically significant difference compared to the control non-vaccinated group (*p ˂ 0.05, ** *p* ˂ 0.01, *** *p* ˂ 0.001 and **** *p* ˂ 0.0001). (**b**) Heat map of fold changes generated by comparing Mx1 expression in vaccinated groups to non-vaccinated group. The fold change is shown on a log_2_ scale, as shown beside the heat map and the different vaccine letter indicate different vaccinated group.

For chIFN-alpha **(**Fig. 4a, b**)**, groups vaccinated with vaccines A, B, D, and E showed increases in cytokine expression at 48 hpv and 3 dpv, ranging from 1.5- to 2.2-fold and 2- to threefold increases, respectively, compared to the non-vaccinated group **(**Fig. 4a, b**)**. This upregulation persisted for up to one week after inoculation and was statistically significant (*p* < 0.05) compared to the control non-vaccinated group. Regarding the ISG (Mx1) **(**Fig. 5a, b**)**, Mx1 transcript levels increased at 3 dpv in birds vaccinated with vaccine A (2.5-fold increase) and vaccine B (2.9-fold increase) compared to the non-vaccinated group **(**Fig. 5a, b**)**. One week after immunization, all vaccinated groups exhibited higher levels of Mx1 transcripts than the non-immunized group **(**Fig. 5a, b**)**.

#### Effect of different commercially available inactivated H9N2 vaccines on the kinetics of chicken MHC-1 and MHC-2

**Fig. 6:**
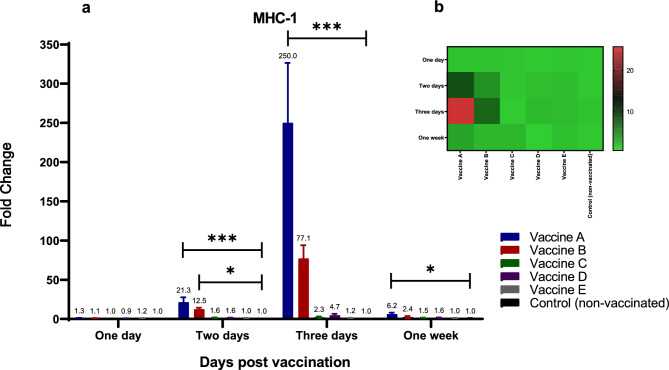
Effect of different commercially available inactivated H9N2 vaccines on the kinetics of chicken MHC-1. Five different commercial AIV (H9) inactivated vaccines were administrated to different experimental groups 4 days of age. Five birds from each group were humanly euthanized 24 h, 48 h, 72 h and one-week post vaccination and their spleen tissues were collected. RNA was extracted and MHC-1 specific quantitative real-time PCR was performed. All data were normalized to GAPDH as an endogenous control and all data were expressed as mean of fold difference relative to non-AIV (H9) vaccinated group, which is given an arbitrary value of 1. (**a**) Error bars represent the standard errors of means and One-way ANOVA was used for the statistical analysis with Dunnett’s test at *p* < 0.05 using GraphPad Prism version 8 for Windows, GraphPad Software, La Jolla California USA, www.graphpad.com. Asterisks indicate the statistically significant difference compared to the control non-vaccinated group (*p ˂ 0.05, ** *p* ˂ 0.01, *** *p* ˂ 0.001 and **** *p* ˂ 0.0001). (**b**) Heat map of fold changes generated by comparing MHC-1 expression in vaccinated groups to non-vaccinated group. The fold change is shown on a log_2_ scale, as shown beside the heat map and the different vaccine letter indicate different vaccinated group.

**Fig. 7:**
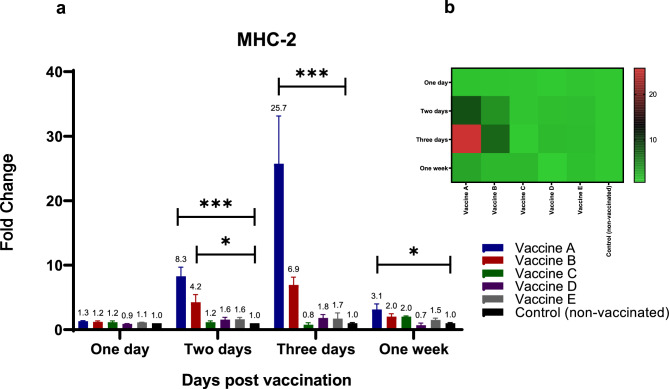
Effect of different commercially available inactivated H9N2 vaccines on the kinetics of chicken MHC-2. Five different commercial AIV (H9) inactivated vaccines were administrated to different experimental groups 4 days of age. Five birds from each group were humanly euthanized 24 h, 48 h, 72 h and one-week post vaccination and their spleen tissues were collected. RNA was extracted and MHC-2 specific quantitative real-time PCR was performed. All data were normalized to GAPDH as an endogenous control and all data were expressed as mean of fold difference relative to non-AIV (H9) vaccinated group, which is given an arbitrary value of 1. (**a**) Error bars represent the standard errors of means and One-way ANOVA was used for the statistical analysis with Dunnett’s test at *p* < 0.05 using GraphPad Prism version 8 for Windows, GraphPad Software, La Jolla California USA, www.graphpad.com. Asterisks indicate the statistically significant difference compared to the control non-vaccinated group (*p ˂ 0.05, ** *p* ˂ 0.01, *** *p* ˂ 0.001 and **** *p* ˂ 0.0001). (**b**) Heat map of fold changes generated by comparing MHC-2 expression in vaccinated groups to non-vaccinated group. The fold change is shown on a log_2_ scale, as shown beside the heat map and the different vaccine letter indicate different vaccinated group.

Increases in MHC-1 and MHC-2 mRNA expression levels were observed as early as 48 h following vaccination with the various vaccines **(**Figs. 6 and 7a, b**)**. The highest levels of MHC-1 were detected after immunization with vaccines A (21-fold) and B (12.5-fold) at 48 h post-vaccination, showing statistically significant differences (*p* < 0.05) compared to the control group **(**Fig. 6a, b**)**. Twenty-four hours later, cytokine levels following immunization with vaccines A (250-fold) and B (77-fold) were significantly higher than those observed after immunization with vaccines C (2.3-fold) and D (4.6-fold), relative to the non-vaccinated group **(**Fig. 6a, b**)**.

MHC-2 levels were highest 48 h following immunization with vaccines A (8.2-fold increase) and B (4.2-fold increase) (Fig. 7a, b). Twenty-four hours later, MHC-2 levels were even higher in birds vaccinated with vaccine A (25.7-fold increase) and vaccine B (sevenfold increase) compared to non-vaccinated birds (Fig. 7a, b). One week after vaccination, groups immunized with vaccines A, B, or C exhibited increases 2 to 3 times greater than those in the non-vaccinated group (Fig. 7a, b).

#### Serological responses conferred by different commercially available inactivated AIV (H9N2) vaccines

**Fig. 8:**
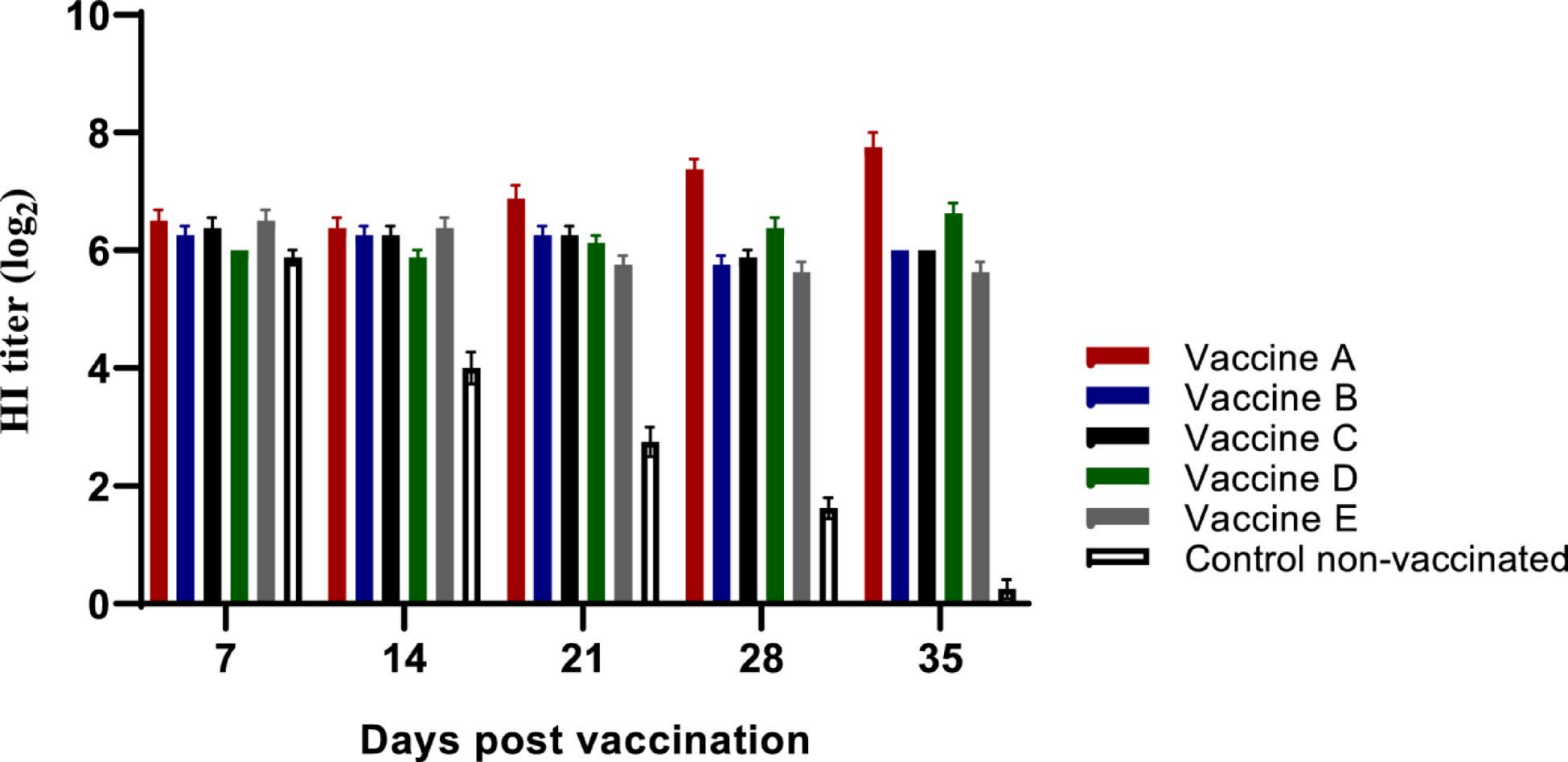
Serology of the experimental birds as determined by the HI test. Serological response of birds vaccinated with different AIV (H9N2) inactivated vaccines, measured using HI test to determine the level of specific antibodies against the applied inactivated vaccines in the different experimental groups. The titers are expressed as GMT ± SE.

The HI test was performed, and a geometric mean titre > 4 log_2_ following vaccination was considered to be protective. The HI test was performed using inactivated AIV H9N2 antigen of G1 lineage (A/chicken/Egypt/FAO-S33/2021(H9N2)) to predict vaccine protection. The maternal derived antibody was declined to ≤ 4 log_2_ at 18–25 days old in non-vaccinated group. As shown in Fig. 8 and Table 3, single vaccination with either of different commercial inactivated H9N2 AI vaccine at 4 days of age led to an AI-HI Ab response > 4 log_2_ from 2 to 5 weeks post vaccination. Single injections of either of the applied vaccines led to AI-HI-Ab responses (5.88–6.38 log_2_) at 14 days post vaccination with statistical significance (*p*˂0.05) difference compared with the non-vaccinated groups. Three, fourth and fifth weeks post vaccination, the AI-HI-Ab responses in the different vaccinated groups were ranged from (5.70—6.88 log_2_), (5.62—7.37 log_2_) and (5.62—7.75 log_2_), respectively. Birds vaccinated with either vaccine A, B or C had no statistical significant differences in the AIV H9N2 geometric mean titers at 3 weeks post vaccination. Fourth and fifth weeks post vaccination birds vaccinated with vaccine A and D had the highest seroconversion against AIV H9N2 compared with the other vaccinated groups (Fig. 8 and Table 3**)**.

**Table 3:** Statistical analysis of HI results following vaccination with different H9N2 vaccines.

Groups	1^st^ week*	2^nd^ week*	3^rd^ week*	4^th^ week*	5^th^ week*
Vaccine A	6.50 ± 0.19^a^	6.37 ± 0.18^a^	6.88 ± 0.23^a^	7.37 ± 0.18^a^	7.75 ± 0.25^a^
Vaccine B	6.25 ± .016^a^	6.25 ± 0.16^a^	6.25 ± 0.16^ab^	5.75 ± 0.16^bc^	6.00 ± 0.00^bc^
Vaccine C	6.38 ± 0.18^a^	6.25 ± 0.16^a^	6.38 ± 0.183^ab^	5.88 ± 0.12^bc^	6.00 ± 0.00^bc^
Vaccine D	6.00 ± 0.00^a^	5.88 ± 0.12^a^	6.12 ± 0.12^b^	6.37 ± 0.18^b^	6.62 ± 0.18^b^
Vaccine E	6.50 ± 0.18^a^	6.38 ± 0.18^a^	5.70 ± 0.16^b^	5.62 ± 0.18^c^	5.62 ± 0.18^c^
Control (non-vaccinated)	5.78 ± 0.13^a^	4.00 ± 0.27^b^	2.75 ± 0.25^c^	1.62 ± 0.18^d^	0.25 ± 0.16^d^

- * Post vaccination.

- Data represented as Mean ± SEM.

- abc Means within the same column carrying different superscripts are sig. different at *p* < 0.05 based on Tukey’s Honestly Significant Difference (Tukey’s HSD) test.

### Study 2: clinical protection of AIV H9N2 vaccinated chickens against 21 days old challenge with AIV H9N2 G1 lineage (A/chicken/Egypt/FAO-S33/2021(H9N2))

#### Clinical protection

The experimental evaluation of the AIV (H9N2) vaccines was expanded in this study using challenge study. Fifteen birds/group were challenged with 10^6^ EID_50_/ml of AIV strain (A/chicken/Egypt/FAO-S33/2021 (H9N2)) via intraocular route. Different clinical outcomes were observed in the experimental groups due to the 21 days exposure to an eye drop infection of the H9N2 AIV strain.

Only 20% (3/15) of the unvaccinated control group died on fourth and fifth day post challenge, compared to 13.3% (2/15) and 6.7% (1/15) mortality rates for the vaccine C and Vaccine D groups, respectively. The remaining experimental groups (Vaccine A, B & E) had a 100% survival rate (Table 4).

**Table 4:** Clinical protection of vaccinated groups against challenge with AIV strain (A/chicken/Egypt/FAO-S33/2021(H9N2)).

Groups	Clinical signs	Days post challenge							Sick % Sick/Total	Clinical protection % Healthy/Total	Mortality % Dead/Total	Protection% Alive/Total
		1	2	3	4	5	6	7				
1^st^ group (Vaccine A)	Normal	15	15	15	13	14	15	15	13.3% 2/15	86.7% 13/15	0.0% 0/15	100% 15/15
	Sick	0	0	0	2	1	0	0				
	Dead	0	0	0	0	0	0	0				
2^nd^ group (Vaccine B)	Normal	15	15	13	11	10	15	15	33.3% 5/15	66.7% 10/15	0.0% 0/15	100% 15/15
	Sick	0	0	2	4	5	0	0				
	Dead	0	0	0	0	0	0	0				
3^rd^ group (Vaccine C)	Normal	15	13	12	8	11	13	13	33.3% 5/15	66.7% 10/15	13.3% 2/15	86.7% 133/15
	Sick	0	2	2	5	2	0	0				
	Dead	0	0	1	1	0	0	0				
4^th^ group (Vaccine D)	Normal	15	13	9	8	11	11	13	40% 6/15	60% 9/15	6.7% 1/15	93.3% 14/15
	Sick	0	2	5	6	3	3	1				
	Dead	0	0	1	0	0	0	0				
5^th^ group (Vaccine E)	Normal	15	15	15	12	11	14	15	26.7% 4/15	73.3% 11/15	0.0% 0/15	100% 15/15
	Sick	0	0	0	3	4	1	0				
	Dead	0	0	0	0	0	0	0				
6^th^ group (control positive)	Normal	15	10	4	3	3	4	5	73.3% 11/15	26.7% 4/15	20% 3/15	80% 12/15
	Sick	0	5	11	10	9	8	7				
	Dead	0	0	0	2	1	0	0				

After challenge, 11/15 of the non-vaccinated birds (73.3%) showed clinical signs of depression, conjunctivitis with closed eye with ocular discharge, and respiratory rales as early as 48 h post challenge (Table 4). The clinical signs lasted up to the 7^th^ days post challenge in survived birds. In the instance of vaccination, the vaccinated birds post challenge showed mild conjunctivitis and mild respiratory signs (Table 4). The lowest clinical signs were recorded in birds vaccinated with vaccine A at 4^th^ days of age (Table 4). The details of the different clinical signs in the different vaccinated groups are listed in **(Supplementary table S1)**.

#### Histopathological changes

**Fig. 9:**
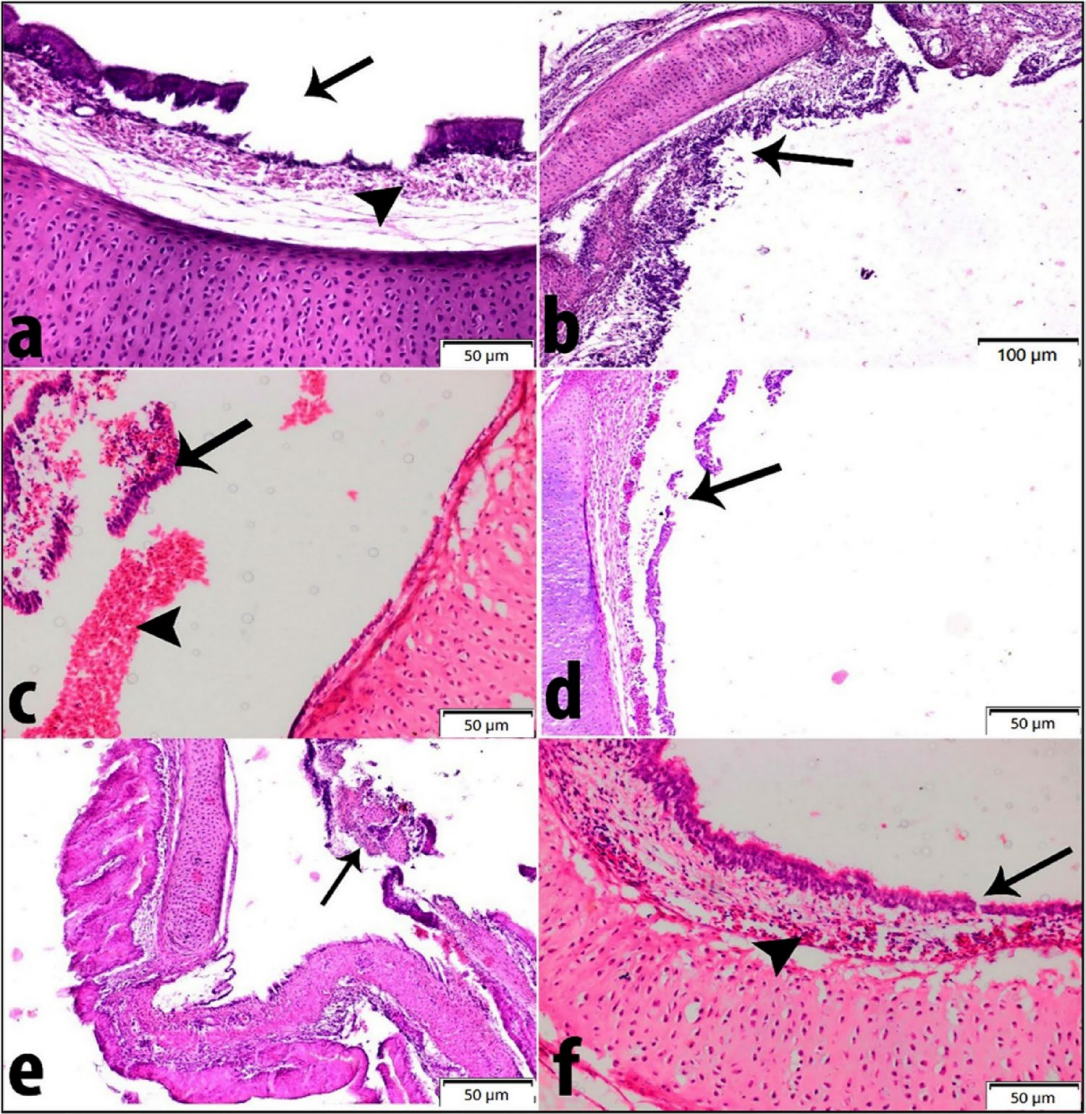
Histopathological changes in bird’s trachea following 21 days old challenge with AIV H9N2 G1 lineage (A/chicken/Egypt/FAO-S33/2021(H9N2)) in AIV H9N2 vaccinated birds. (**a**) Tracheal section of positive control broiler group showing necrosis, sloughing of lining epithelium (arrow) and sub-mucosal hemorrhages (arrow head) (HE, Bar = 50 μm). (**b**) Tracheal section of 1^st^ group (Vaccine A) showing desquamation of lining epithelium with complete loss of cilia (arrow) (HE, Bar = 100 μm). (**C**) Tracheal section from 2^nd^ group (Vaccine B) showing necrosis, sloughing of lining epithelium (arrow) and hemorrhages (arrow head) (HE, Bar = 50 μm). (**d**) Tracheal section from 5^th^ group (vaccine E) showing desquamation of lining epithelium (arrow) and congested blood vessels (HE, Bar = 50 μm). (**e**) Tracheal section from 4^th^ group (Vaccine D) showing severe necrosis, sloughing of lining epithelium and inflammatory cells infiltration (arrow) (HE, Bar = 50 μm). (**f**) Tracheal section from 3^rd^ group (Vaccine C) showing degeneration and sloughing of lining epithelium (arrow) and presence of large number of erythrocytes in submucosa (arrow head) (HE, Bar = 50 μm).

For histopathological examination of trachea, lesions as necrosis, sloughing of lining epithelium, hemorrhages, loss of cilia and inflammatory cell infiltration were observed. The degree of lesion varies from moderate to highly severe as shown in (Fig. 9a, f and Tables 5 and 6). Tracheal section of non-vaccinated challenged control birds had necrosis, sloughing of lining epithelium and sub-mucosal hemorrhages. Tracheal section of birds vaccinated with vaccine A had desquamation of the lining epithelium with complete loss of cilia while birds vaccinated with vaccine B had necrosis, sloughing of lining epithelium and hemorrhages. In the instance of vaccine C, tracheal section showed degeneration and sloughing of lining epithelium and presence of large number of erythrocytes in submucosa. Tracheal section from vaccine D group showed severe necrosis, sloughing of lining epithelium and inflammatory cell infiltration while vaccine E showed desquamation of lining epithelium (arrow) and congested blood vessels. Numerical scoring for the detailed histological tracheal lesions are listed in Table 6.

**Table 5:** Score of pathological lesions in trachea and lung.

	Trachea				Lung			
Groups	Mild	Moderate	Severe	Highly severe	Mild	Moderate	Severe	Highly severe
1^st^ group (Vaccine A)	–	+	–	–	–	+	–	–
2^nd^ group (Vaccine B)	–	–	+	–	–	+	–	–
3^rd^ group (Vaccine C)	–	–	–	+	–	–	+	–
4^th^ group (Vaccine D)	–	–	–	+	–	–	+	–
5^th^ group (Vaccine E)	–	–	+	–	–	+	–	–
6^th^ group (Control positive)	–	–	–	+	–	–	+	–

**Table 6:** Lesions score of the severity extent in trachea and lung among different experimental groups.

Organ	Lesions	1st group (Vaccine A)	2^nd^ group (Vaccine B)	3^rd^ group (Vaccine C)	4^th^ group (Vaccine D)	5^th^ group (Vaccine E)	6^th^ group (Control positive)
Trachea	Necrosis and sloughing of lining epithelium	2	3	4	4	3	4
	Submucosal hemorrhages	1	3	4	4	2	4
	Inflammatory cells infiltration	2	3	3	3	3	4
Lung	Necrosis and sloughing of lining bronchiolar epithelium	2	2	3	3	2	3
	Congestion of pulmonary vessels	2	2	3	3	2	3
	Inflammatory cells infiltration	1	1	2	3	2	3
	Deposition of fibrinous exudate (fibrinous pneumonia)	1	2	3	2	1	3

Examined chickens = 3 chickens /group. Number of examined fields (3 random fields/group). The lesions were graded by estimating by semiquantitative methods “Ordinal Method “in the entire sections. Lesions score system was as follows: 0 = absence of lesion, 1 = mild, 2 = moderate, 3 = severe and 4 = highly severe.

**Fig. 10:**
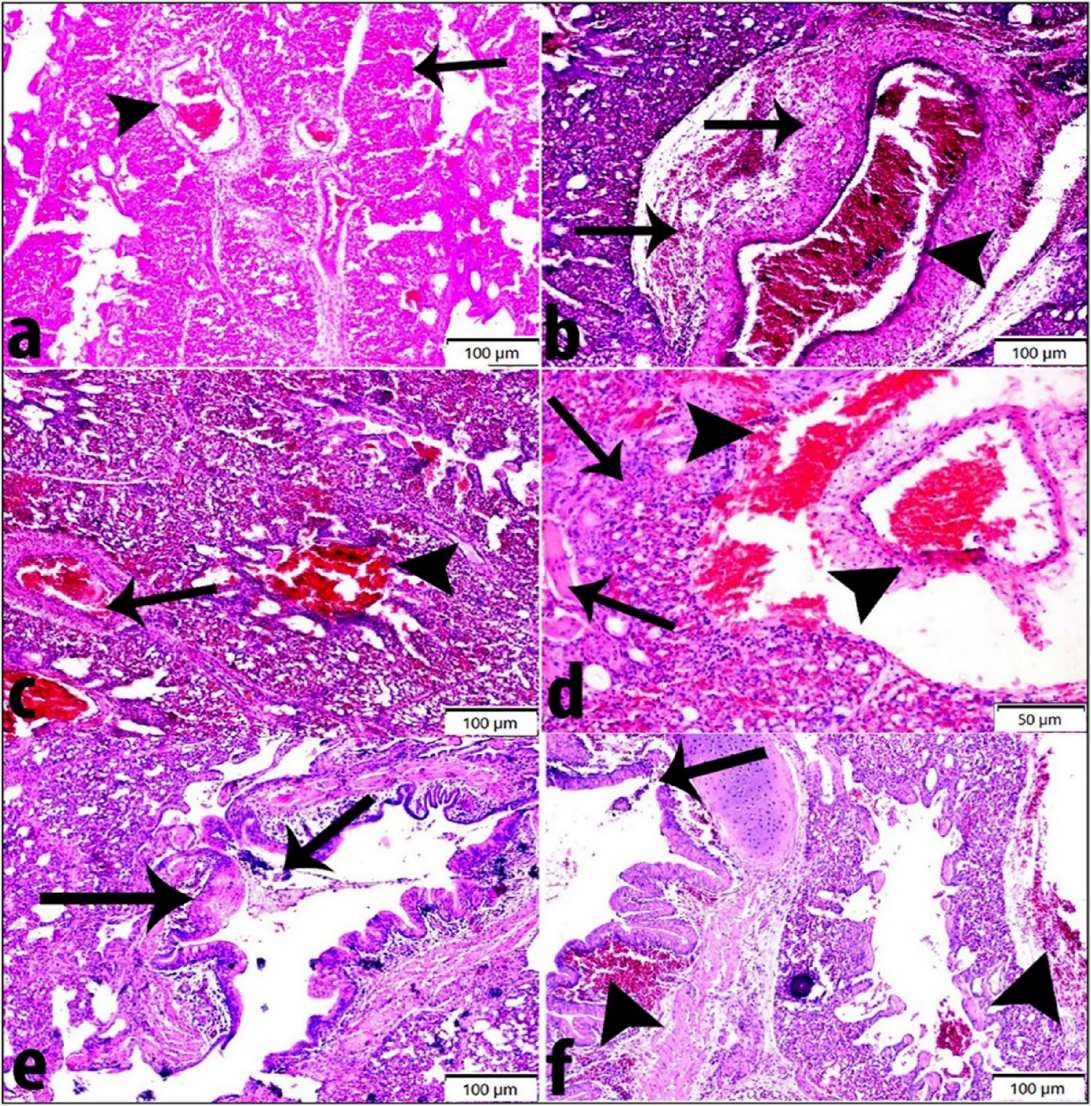
Histopathological changes in bird’s lung following 21 days old challenge with AIV H9N2 G1 lineage (A/chicken/Egypt/FAO-S33/2021(H9N2)) in AIV H9N2 vaccinated birds. (**a**) Lung section of positive control broiler group showing congestion of pulmonary vessels (arrowhead) and deposition of fibrinous exudate (fibrinous pneumonia) (arrow) (HE, Bar = 100 μm). (**b**) Lung from 1^st^ group (Vaccine A) showing congested pulmonary vessel, perivascular edema lymphocytic cells infiltration and thickening of blood vessel walls with deposition of fibrinous materials (arrow) (HE, Bar = 100 μm). (**C**) Lung from 2^nd^ group (Vaccine B) showing congested blood vessel (arrow) and hemorrhages (arrowhead) (HE, Bar = 100 μm). (**d**) Lung section from 5^th^ group (Vaccine E) showing deposition of fibrinous exudate, inflammatory cells infiltration (arrow), and congested blood vessels with perivascular hemorrhages (arrowhead) (HE, Bar = 50 μm). (**e**) Lung section from 4^th^ group (Vaccine D) showing necrosis, sloughing of lining bronchiolar epithelium and inflammatory cells infiltration (arrow) (HE, Bar = 100 μm). (**f**) Lung from 3^rd^ group (Vaccine C) showing degeneration and sloughing of lining epithelium of bronchi (arrow) and presence of large number of erythrocytes in submucosa and surrounding alveolar tissue (arrow head) (HE, Bar = 100 μm).

For examination of lung tissue under light microscope, histopathological changes as congestion of pulmonary vessels, deposition of fibrinous exudate, sloughing of lining epithelium, perivascular edema and inflammatory cell infiltration were observed. The degree of lesion varies from moderate to severe as shown in (Fig. 10a, f and Tables 5 and 6). Lung section of positive control broiler group showing congestion of pulmonary vessels and deposition of fibrinous exudate (fibrinous pneumonia). Lung from birds vaccinated with vaccine A showing congested pulmonary vessel, perivascular edema lymphocytic cell infiltration and thickening of the blood vessel walls with deposition of fibrinous materials. Lung from vaccine B immunized group showing congested blood vessel and hemorrhage. Lung from vaccine C immunized group showing degeneration and sloughing of lining epithelium of bronchi and presence of large number of erythrocytes in submucosa and surrounding alveolar tissues. Lung section from vaccine D immunized group showing necrosis, sloughing of lining bronchiolar epithelium and inflammatory cell infiltration. Lung section from vaccine E immunized group showing deposition of fibrinous exudate, inflammatory cell infiltration and congested blood vessels with perivascular hemorrhages. Numerical scoring for the detailed histological lung lesions are listed in Table 6**.**

## Discussion

Avian influenza (AI) is a contagious viral respiratory disease of poultry caused by any subtype (H1–H18, N1–N11) of type A influenza virus. It results in significant losses to the poultry industry and poses risks to human health^29–31^. Avian influenza viruses belong to the Orthomyxoviridae family and are characterized by a single-stranded, negative-sense RNA genome segmented into eight parts, encoding ten distinct proteins^32^. It can be classified into two categories based on pathogenicity: highly pathogenic avian influenza virus "H5/H7" (HPAIV) and low pathogenic avian influenza virus (LPAIV)^3,31^. Since 2011, the LPAI H9N2 subtype has been identified in domestic quails in Egypt before becoming widespread in domestic poultry^33^. The H9N2 avian influenza virus has spread extensively among poultry, causing substantial economic losses, especially when secondary infections with other pathogens occur. Importantly, H9N2 AIV continues to infect humans, and its six internal genes frequently reassort with those of other influenza viruses, leading to novel influenza strains that infect humans and pose a public health risk. Biosecurity measures and stringent vaccination programs are essential components of the prevention and control strategy for H9N2 AIV. Inactivated whole-virus vaccines have been predominantly used to control H9N2 AIV. However, H9N2 AIVs may still be detected in immunized poultry flocks and have recently emerged as the primary epidemic subtype^6^. A more effective and modern vaccine prevention strategy may be necessary to address the current situation^34^.

Immune defense against AIV is primarily mediated by both humoral and cellular immune responses^35^. Humoral antibodies targeting hemagglutinin and neuraminidase (NA) antibodies are used to be key indicators of protection for traditional influenza vaccines^36,37^. However, studies have shown that protection against AIV does not always correspond directly with the strength of the humoral immune response^38,39^. Therefore, a thorough comparative study along with an understanding of the underlying immunological mechanisms and innate immune profile elicited post vaccination has become necessary. The innate immune system is considered the body’s first line of defense, capable of combating pathogens immediately upon their entry. It consists of physical and chemical barriers, innate immune cells, including dendritic cells, natural killer (NK) cells, and macrophages, pattern recognition receptors on innate immune cells, and complement proteins and cytokines. Unlike adaptive immunity, innate immunity responds instantly to pathogens and is the first to be activated within the body.

In this study, the protective effects and immunological responses of five distinct commercially available inactivated AIV (H9N2) whole virus vaccines with varying vaccine seeds and adjuvant strategies were investigated. The phenotypic changes in both innate and adaptive immune responses following vaccination were analyzed. An earlier study reported that the H7N9 LPAIV induces specific local and systemic cellular immune responses in chickens when administered via the natural intranasal route^40^. Thereby, the data in the aforementioned report indicated that KUL01 + , MHC-2 + monocytes/macrophages, putative DC, and CD3 − CD8α + NK participate in host defense against the low pathogenic H7N9 infection. Additionally, infection with H9N2 AIV, whether live or inactivated, induces strong innate antiviral and inflammatory responses involving TLR3, TLR7, MDA5, TNF-α, and CCL5 stimulation^41,42^.

Adjuvants may have an essential role as a consistent delivery system for the inactivated vaccine and contributing role as enhancer to the innate-cellular or mucosal immunity^43,44^. Incorporating pathogen-associated molecular patterns (PAMPs) into vaccine adjuvants is a novel strategy to enhance both innate and subsequent adaptive immune responses. Pathogen-associated molecular patterns are molecular motifs, such as viral protein, single-stranded RNA, double-stranded RNA, or CpG island DNA, that provide signatures recognized by PRR on immune system cells as well as epithelial cells from body barrier sites. The membrane-bound toll-like receptor system is one of the most well-known PRR systems. The endosome-resident TLR-7 may detect viral RNA from live or inactivated influenza viruses and employ various adaptor proteins to send signals that cause splenocytes, plasmacytoid dendritic cells, and/or HD11 cells to produce chemokine, inflammatory cytokines as (IL-6) and antiviral cytokines as chIFN-alpha^45^. On the other hand, the cell surface-resident TLR-5 can be stimulated via pathogen components such as bacterial flagellin, and activates the NF-κB through MyD88 adaptor protein to promote the induction of proinflammatory cytokines, not antiviral, such as (IL-6)^46–48^. The incorporation of CpG as an adjuvant in the inactivated H9N2 AIV vaccine enhanced the overall efficacy of the vaccination^49,50^. Consequently, dendritic cells responded rapidly by maturing and activating the TLR signaling pathway. This activation was accompanied by the upregulation of co-stimulatory molecules (CD40, CD80, CD86, and MHC-II), regulatory proteins (IRF-7 and TRAF-6), and pro-inflammatory cytokines (IL-1, IL-6, and IL-12).

In this study, quantitative real-time PCR analysis demonstrated that distinct inactivated H9N2 AIV vaccines, formulated with different vaccine strains and emulsified adjuvants, stimulated both PRRs, TLR-5 and TLR-7. Following the PRRs upregulation, the expression of chIFN-alpha, Mx1, and IL-6 served as indicators of the downstream effects of this signaling cascade. Activation of TLR-7 was detected as early as 48 hpv, with elevated levels persisting up to one week post-vaccination in the cases of vaccines A and B. Elevation of TLR-7 and MDA5 in the oviducts has also been observed in laying hens infected with H9N2^51^. Furthermore, vaccines A, B, and E induced a more pronounced upregulation of TLR-5 mRNA levels at 2 and 3 dpv; however, this elevation remained robust at 7 dpv only in birds vaccinated with vaccine A. Although TLR5 is typically activated by bacterial flagellin, AIV can also trigger TLR5 activation in certain contexts. This may be due to the presence of flagellin-like structures or the inclusion of TLR5 agonists, such as flagellin used as an adjuvant, which can enhance the immune response to avian influenza vaccines and potentially reduce viral replication^52–54^.

The detection of viral nucleic acids by TLRs initiates signaling pathways that lead to the production of type I interferons in chickens. Chicken interferon-alpha, a type I interferon, is a potential inhibitory and therapeutic antiviral agent that suppresses H9N2. This type I interferon signaling subsequently promotes the transcription of various interferon-stimulated genes (ISGs), including Mx1, which play essential roles in antiviral defense mechanisms^22,23^. Furthermore, type I interferon signaling is closely linked to the production of the T-helper 2 (Th2) pro-inflammatory cytokine (IL-6). Interleukin-6 acts as a critical mediator in adaptive immune responses, particularly by supporting the generation and maturation of B cells in the context of AIV-induced inflammatory processes^55,56^.

Consistent with the activation of the studied TLRs, the kinetics of interferon-alpha mRNA expression in spleen cells in response to various vaccines exhibited patterns similar to those previously reported^57^. Furthermore, stimulation of the two studied PRRs in all immunized groups with different inactivated H9N2 vaccines resulted in increased IL-6 mRNA levels in chicken spleens to varying degrees, beginning as early as 48 hpv and lasting up to one week post-vaccination. Interleukin-6 promotes T cell activation and differentiation, as well as antibody production, by stimulating B cells^58,59^. In consistent, the elicitation of the IL-6 mRNA levels were exteremly evident one-week pv as indicator for the initiation of the adaptive immune responses.

Stimulation of PRRs triggers the maturation of dendritic cells, stabilizing major histocompatibility complex molecules on their surfaces. This process also enhances antigen presentation by increasing the expression of costimulatory antigen presenting cells (APCs), leading to T cell proliferation and differentiation. Therefore, activating PRRs benefits the host in two primary ways: it first initiates innate immune responses to directly eliminate pathogens, and if these responses are insufficient, it activates antigen-specific adaptive immune responses to provide an additional level of defense^60,61^.

As a consequence, the mRNA expression levels of both MHC-I and MHC-II were monitored over one week following vaccination. Three days following vaccination, MHC-1 mRNA levels increased by up to 250 fold and 77 fold for vaccines A and B, respectively. MHC-2 levels were highest for vaccines A (8.2–5.7 fold) and B (4.2–sevenfold) 48 h and 3 days after immunization, respectively. The substantial expression of MHC molecules, together with the conspicuous expression of cytokines such as IL-6, may indicate the stimulation of CD4 + T helper cells and the early onset of adaptive immune responses in vaccinated groups, particularly birds immunized with vaccine A and B.

In accordance with the previous, a single vaccination with different commercial inactivated H9N2 AI vaccines at 4 days of age resulted in an AI-HI Ab responses > 5 log_2_ from 2 to 5 weeks following vaccination, with privilege in the titers induced by vaccine A. The previous may be attributed to the increases in MHC-1 & 2, and IL-6 mRNA levels, which resulted in an increase in bird survival and minimizing of the histopathological lesion scores in trachea and lung of birds vaccinated with inactivated vaccine with PAMP emulsified adjuvant following AIV (H9N2) challenge. However, it is worth mentioning that all tissue samples subjected to histopathological examination tested positive for virus detection using RT-PCR analysis (data not shown).

According to a recent study, PAMP immunization reduced the histopathological lesions in chickens vaccinated with LaSota when compared to their counterparts who were not vaccinated^62^. This might be because the PAMP immunization increased both innate and adaptive immunity, as evidenced by the serological responses. Notably, both vaccinations B and E produced 100% clinical protection and reduced respiratory pathological damage in response to the challenge, and both vaccines have virus seed, which are phylogenetically similar to the challenge strain.

**In conclusion:** LPAI (H9N2) vaccines able to stimulate the expression of different pattern recognition receptors as well as their downstream cytokines involved in pro-inflammatory, anti-viral, and cell-mediated and adaptive immune responses. H9N2 inactivated vaccines able to produce AIV (H9N2) HI-Ab protective titers 2–5 weeks post vaccination. Pathogen associated molecular patterns as adjuvant has a beneficial effect in inactivated vaccine immunology and improve bird protection as well as minimize the respiratory system histopathological scores post challenge with AI (H9N2) virus. This research offers a new immunological perspective on H9N2 vaccinations.

## Supplementary Information

Supplementary Information.

## Data Availability

The authors confirm that the data supporting the findings of this study are available within the manuscript, figures, and tables.
