# Supplementary material for: The impact of innate and humoral immune mechanisms on vaccine induced protection against avian influenza H9N2 in broilers
Source: Sci Rep. 2025 Dec 2;15:43016. doi: 10.1038/s41598-025-28122-2 (PMC12675785; doi:10.1038/s41598-025-28122-2)
Supplement: Supplementary file 1 — Supplementary Information. [file 41598_2025_28122_MOESM1_ESM.docx]

Table S1: Clinical signs and Mortality in different vaccinated and non-vaccinated groups following challenge with AIV strain (A/chicken/Egypt/FAO-S33/2021(H9N2)) at 14 days-old.

| **Challenge** | **Groups** | **Conjunctivitis** | **Respiratory rales** | **ocular discharge** | **Nervous signs** | **Mortality** |
| --- | --- | --- | --- | --- | --- | --- |
| 14 days old | 1^st^ group (Vaccine A) | 2/15 | 1/15 | 1/15 | 0/15 | 0/15 |
|  | 2^nd^ group (Vaccine B) | 4/15 | 2/15 | 3/15 | 0/15 | 0/15 |
|  | 3^rd^ group (Vaccine C) | 5/15 | 3/15 | 4/15 | 0/15 | 2/15 |
|  | 4^th^ group (Vaccine D) | 6/15 | 3/15 | 5/15 | 0/15 | 1/15 |
|  | 5^th^ group (Vaccine E) | 4/15 | 2/15 | 3/15 | 0/15 | 0/15 |
|  | 6^th^ group (control positive) | 10/15 | 6/15 | 8/15 | 0/15 | 3/15 |
